# Reply to letter on What biological markers could be used for diagnosis and monitoring of nitrous oxide abuse

**DOI:** 10.1111/ene.16187

**Published:** 2023-12-20

**Authors:** Etienne Fortanier, Edouard Berling, Adrien Zanin, Adrien Le Guillou, Joelle Micaleff, Guillaume Nicolas, Pierre Lozeron, Shahram Attarian

**Affiliations:** ^1^ Reference Center for Neuromuscular Diseases and amyotrophic lateral sclerosis La Timone University Hospital, Aix‐Marseille University Marseille France; ^2^ APHP, Service de Neurologie, Hôpital Raymond Poincaré, Centre de Référence Nord‐Est‐Ile‐de‐France FHU PHENIX Garches France; ^3^ Université de Versailles Saint‐Quentin‐en‐Yvelines U1179 INSERM Paris‐Saclay France; ^4^ Service de Physiologie Clinique‐Explorations Fonctionnelles, DMU DREAM, APHP Hôpital Lariboisière Paris France; ^5^ Laboratory for Vascular Translational Science, INSERM, U1148 Université de Paris Paris France; ^6^ Department of Epidemiology Emory University Atlanta Georgia USA; ^7^ Marseille University Hospital, Clinical Pharmacology and Pharmacosurveillance Regional Addictovigilance Center of Marseille Marseille France; ^8^ INSERM UMR1106 Aix‐Marseille University Marseille France; ^9^ INSERM, GMGF Aix‐Marseille University Marseille France

Dear Editor,

We would like to thank you for the opportunity to respond to the remarks presented in the letter “What biological markers could be used for diagnosis and monitoring of nitrous oxide abuse?” We would also like to thank Gernez et al. for their interest and contribution to our article [1].

The authors proposed some suggestions about the different biological biomarkers in nitrous oxide (N_2_O) intoxication by highlighting the relevance of homocysteine and methylmalonic acid (MMA) over serum vitamin B12 measurement.

The primary objective of our study was to help clinicians to rapidly discriminate between N_2_O‐induced neuropathy (N_2_On) and its main differential diagnosis, namely, Guillain–Barré syndrome [2], by characterizing the most efficient clinical, biological, and electrophysiological biomarkers. As mentioned by the authors, the diagnosis is mainly based on discriminating clinical features, and we tried to describe the typical N_2_On patient in the first part of the Results section. However, as detailed in the article, the diagnosis can be challenging, with equivocal clinical presentations and hidden N_2_O misuse at first interview, requiring the analysis of biological and electrophysiological biomarkers.

With regard to biological biomarkers, vitamin B12 serum level was reduced in only half of our patients, as reported recently in the literature [3]. Nevertheless, the use of a slightly different threshold presented a good sensitivity and specificity to distinguish between the two groups of patients, which was the purpose of our work.

We agree with the authors' observations on the relevance of homocysteine and MMA in N_2_O intoxication. However, as mentioned in the article, the average time to obtain these results in our three centers was approximately 7 days, making it difficult to use them as differential biomarkers in this context.

MMA has been correlated to the clinical severity of N_2_O intoxication in a previous study [4]. In our cohort, there was no correlation between homocysteine or MMA serum levels and clinical severity. This may be due to some missing data concerning MMA serum levels, resulting in a lack of statistical power. Larger national cohorts will undoubtedly enable us to better assess the correlation between biological and clinical biomarkers in the future.

Finally, we agree with the authors in their recommendation to perform a combination of biological tests (at least vitamin B9 and B12, homocysteine, and MMA) as quickly as possible, another issue being the fairly rapid correction of some markers after vitamin supplementation, which is sometimes carried out in emergency departments before any blood testing.

## AUTHOR CONTRIBUTIONS

**Etienne Fortanier:** Conceptualization; writing – review and editing; writing – original draft. **Edouard Berling:** Writing – review and editing. **Adrien Zanin:** Writing – review and editing. **Adrien Le Guillou:** Writing – review and editing. **Joelle Micaleff:** Writing – review and editing; supervision. **Guillaume Nicolas:** Supervision; writing – review and editing. **Pierre Lozeron:** Supervision; writing – review and editing. **Shahram Attarian:** Supervision; writing – review and editing.

## CONFLICT OF INTEREST STATEMENT

None of the authors has any conflict of interest to disclose.

## Data Availability

The data that support the findings of this study are available from the corresponding author upon reasonable request.
